# Thermochemically Stable Novel Oxygen Carriers Based on CaMn_1–*x*–*y*_Ti_*x*_Fe_*y*_O_3−δ_ for Chemical Looping

**DOI:** 10.1021/acs.energyfuels.4c02625

**Published:** 2024-07-26

**Authors:** Zuoan Li, Yngve Larring

**Affiliations:** Sustainable Energy Technology, SINTEF, P.O. Box 124, NO-0314 Oslo, Norway

## Abstract

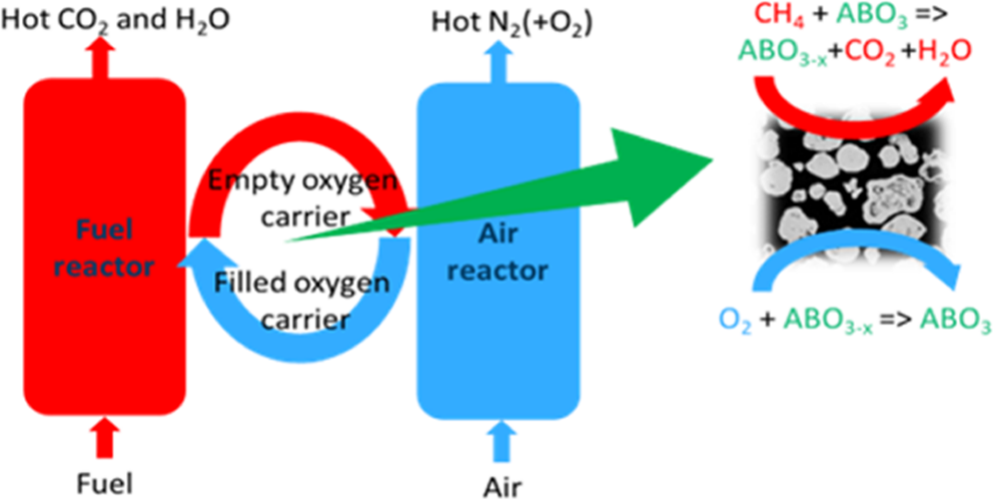

The understanding and development of stable redox materials based on cheap and abundant elements, forming Ca–Mn–Ti–Fe–O-based perovskites, have been in focus for applications in renewable technologies such as chemical looping combustion and thermal energy storage. The present research focuses on developing stable materials to be utilized up to 1050 °C in a CLC process and has shown that the structure stability and oxygen transfer capacity can be achieved by tuning the content of different elements on B-sites of the perovskites. Various experiments, such as redox cycling under various fuels, temperatures, and *p*O_2_, were carried out to evaluate the oxygen transfer capacity, reaction rates under various fuels, etc. The redox stability at high temperatures was evaluated by redox cycles at 1050 °C followed by post SEM analyses on surface and depth profiling. The three developed materials can avoid phase change during redox due to the moderate oxygen transfer capacity of up to 5.6 wt % O_2_ for CaMn_0.5_Ti_0.375_Fe_0.125_O_3−δ_ at 1050 °C, which is important for having stable particles. Cation diffusion was also investigated during redox cycling in the development of stable redox materials, and only a minor diffusion of Mn to the grain boundaries is seen in the least stable material. The findings show that perovskites with high stability can be obtained with more Ti on B-sites, termed as CaMn_0.375_Ti_0.5_Fe_0.125_O_3−δ_. The developed stable oxides, to some extent, have a reduced activity compared to the less stable composition with less Ti and more Mn, termed as CaMn_0.5_Ti_0.375_Fe_0.125_O_3−δ_, which possesses a higher oxygen release to inert ca. 1.1 wt % O_2_ compared to more stable CaMn_0.375_Ti_0.5_Fe_0.125_O_3−δ_ that can release up to 0.8 wt % O_2_. Two of the materials have faster kinetics than ilmenite by a factor of 2 in H_2_.

## 1 Introduction

Concerns over global climate change resulting from fossil fuels, plastic waste, solid recovered fuels, biomass, and biogas necessitate the development of new power generation technologies with CO_2_ capture.^1−3^ Chemical looping combustion (CLC) has emerged as a promising technology with a carbon capture potential near to 100%. This is achieved by cyclic redox reactions of an oxygen carrier material (OCM) in two different reactors: a fuel and an air reactor. In the fuel reactor, the carbonaceous fuel is combusted with oxygen from the OCM to CO_2_ and H_2_O, yielding a pure CO_2_ stream after condensation.^4,5^ In the air reactor, the reduced OCM is reoxidized to produce heat for power generation and chemicals.

Although CLC is flexible for the combustion of different fuels, application of this technology has been hindered by major challenges related to particularly required properties for the OCMs, including a high oxygen transfer capacity (OTC) and catalytic reactivity toward various fuels, thermomechanical strength, and a stable long-term performance over many redox cycles, in addition to being cost-effective and environmentally friendly. Degradation can often result in a temporary good performance related to large available surface areas before complete breakdown. This has often been observed in minerals such as Fe or Mn ores, which are proven unsuitable as candidate OCMs since they lose activity during redox cycles or are prone to agglomeration and fragmentation at high temperatures.^6^ Therefore, it is of great importance to design long-term stable and low-cost OCMs that can keep a stable and predictable performance from the very first redox cycle, i.e., without activation.

Perovskite-type ceramic oxides (ABO_3_) have attracted a lot of attention in renewable energy-related fields such as solid oxide fuel cell (SOFC) cathodes, catalysts, and oxygen transport membranes.^7,8^ The potential advantages of using perovskite-structured materials are their abilities to tolerate a large range of dopants on either the A- or B-site, enabling tailoring of their properties for specified applications. The materials can be compositionally tuned to control the oxygen vacancy concentration and mobility of oxide ions and hence the oxygen transfer capacity and stability in different ambient atmospheres and temperatures. Calcium manganite partially substituted with titanium and/or iron has shown great promise for CLC applications.^9−14^ In order to develop even more stable OCMs with a capable performance, we chose the compositions by considering Ti-rich for structure stabilization and Mn-rich for high reactivity, in addition to Fe to adjust the symmetry of the perovskites, as well as increased hardness. By using thermogravimetry, scanning electron microscopy (SEM), and *p*O_2_ stepping, we have performed detailed investigations toward the OTC and chemical stability under different redox cycles and oxygen transport properties at elevated temperatures of three compositions CaMn_0.375_Ti_0.5_Fe_0.125_O_3−δ_ (termed as CMTF8341), CaMn_0.5_Ti_0.5_Fe_0_O_3−δ_ (CMTF8440), and CaMn_0.5_Ti_0.375_Fe_0.125_O_3−δ_ (CMTF8431).

## 2 Experimental Section

### 2.1 Sample Preparation

Ceramic powders with nominal compositions CaMn_0.375_Ti_0.5_Fe_0.125_O_3−δ_ (CMTF8341), CaMn_0.5_Ti_0.5_Fe_0_O_3−δ_ (CMTF8440), and CaMn_0.5_Ti_0.375_Fe_0.125_O_3−δ_ (CMTF8431) were synthesized by a solid-state reaction, i.e., simple milling and mixing of raw oxide powders followed by calcination at 600 °C for 5 h. Ceramic disc samples (⌀ = 13 and 21 mm) were prepared by uniaxial cold pressing at approximately 85 MPa and the samples were sintered at 1300 °C in ambient air for 6 h with heating and cooling rates of 180 °C h^–1^ to achieve a relative density of above 95% of the theoretical value as measured by Archimedes’ method.

For redox cycling measurements, the dense samples were crushed and sieved through meshes between 180 and 300 μm. For the stability measurement, the disc sample with ⌀ = 13 mm was polished down to a 3 μm surface roughness with SiC grinding paper and subsequently with a diamond abrasive.

### 2.2 Oxygen Transfer Capacity vs Partial Pressure of Oxygen

To determine the real oxygen transfer capacity, the isothermal thermogravimetric experiments were performed on a Setsys Evolution (SETARAM) thermogravimetric analyzer at 950, 1000, and 1050 °C. An isothermal stepping experiment was performed on each sample powder from oxidizing to reducing conditions. Under different oxygen partial pressures, the sample was kept for several hours to reach the thermodynamic equilibrium. The mass loss under each condition was recorded while the real oxygen partial pressures were calculated by FactSage software.

### 2.3 Redox Cycling Using H_2_ Fuel

**Figure 1:**
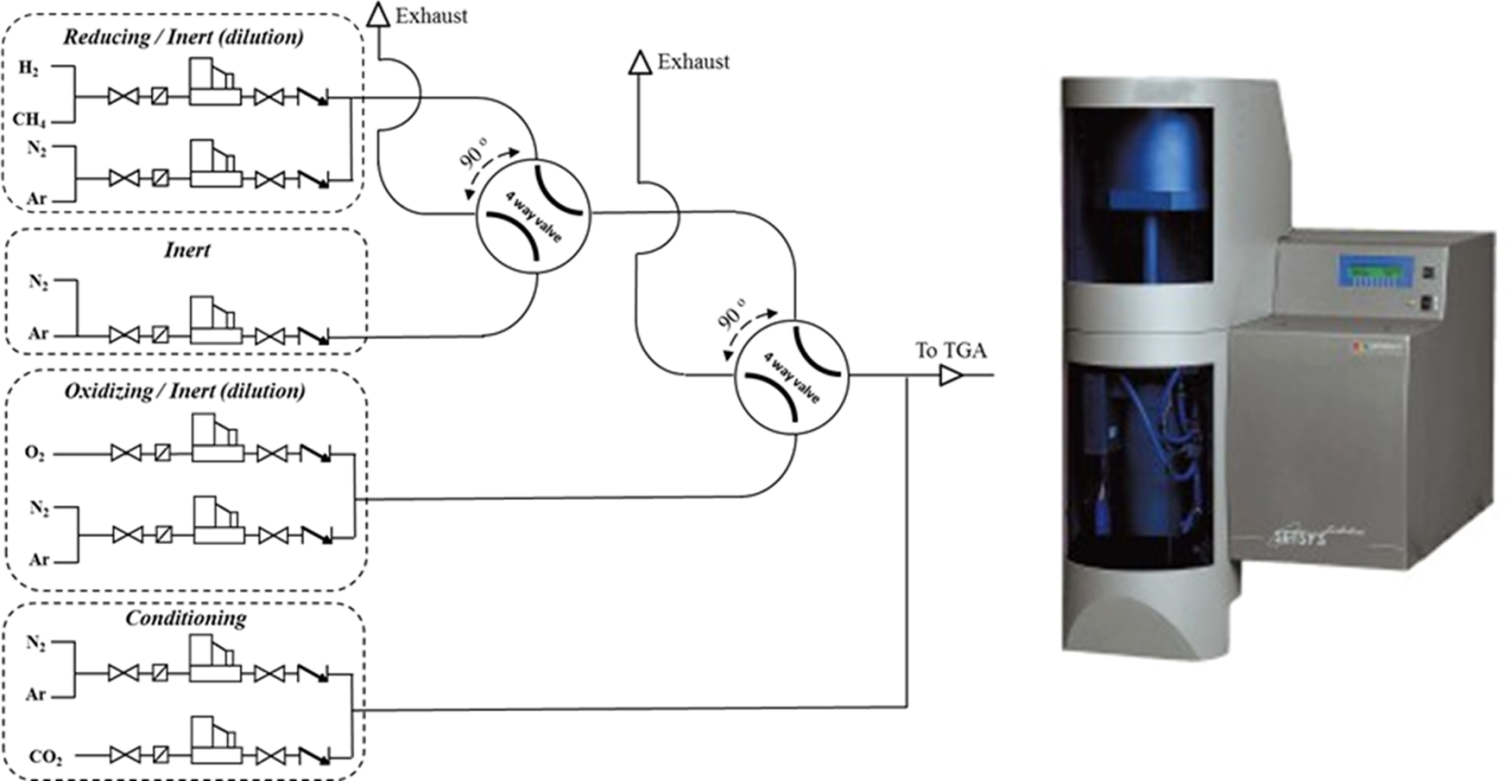
Thermogravimetric analyzer (TGA) with an automatic gas exchange system.

Reduction–oxidation (redox) cycles were performed on a Setsys Evolution (SETARAM) thermogravimetric analyzer apparatus coupled with a fully automated in-house gas mixing system (Figure 1). The materials were first heated to 900 °C in air, and then 20 redox cycles were performed between wet 20% O_2_ and wet 5% H_2_ for activation. Nineteen redox cycles between 10% H_2_ ↔ 20% O_2_, 10% H_2_ ↔ 10% O_2_, 10% H_2_ ↔ 5% O_2_, 5% H_2_ ↔ 20% O_2_, 5% H_2_ ↔ 10% O_2_, and 5% H_2_ ↔ 5% O_2_ were performed at 950, 1000, and 1050 °C. A mix of 25% CO_2_ plus inert Ar with a total flow rate of 200 mL/min was used for conditioning in all of the gas mixtures. The humidity of the reactive gas was controlled via bubbling the gas through a saturated KBr solution at room temperature. During each redox cycle, samples were kept for 2 min in a reducing atmosphere and for 2 min in an oxidizing atmosphere with an inert period of 2 min in between. These conditions were chosen to estimate the apparent OTC for the measured OCM and to give an indication of reversibility after each cycle, the effect of activation, and the redox reaction rate. The mixtures of wet 20% O_2_ + 25% CO_2_ and 5% O_2_ + 25% CO_2_ simulate the conditions close to the inlet and outlet of the air reactor. The wet H_2_/CO_2_ buffer results in a shift reaction, giving milder reducing conditions and a weaker driving force for the reaction, simulating the inlet and outlet of the fuel reactor. The buoyancy effect during redox cycles was determined separately on the system without oxygen carriers, showing a negligible effect for these test conditions. It should also be mentioned here that redox cycles under such conditions are very harsh, and more degradation of OCMs can be expected as compared to normal reactor testing reported in literature.

### 2.4 Redox Cycling Using CH_4_ and CO Fuels

To evaluate the reactivity of oxygen carriers with other fuels including CO and CH_4_, the oxygen carrier was first heated up to 800 °C in wet air, followed by 20 redox cycles between wet air + 25% CO_2_ and wet 5% CO + 25% CO_2_ in inert conditions, and subsequently 20 cycles between wet air + 25% CO_2_ and wet 10% CH_4_ + 25% CO_2_ in inert conditions were performed. The same gas mixing program was used for the temperatures at 850, 900, 950, and 1000 °C. The total flow rate, humidity control, and purging time are the same as those for H_2_ fuel above.

### 2.5 Stability Evaluation under Isothermal Redox Cycling

**Figure 2:**
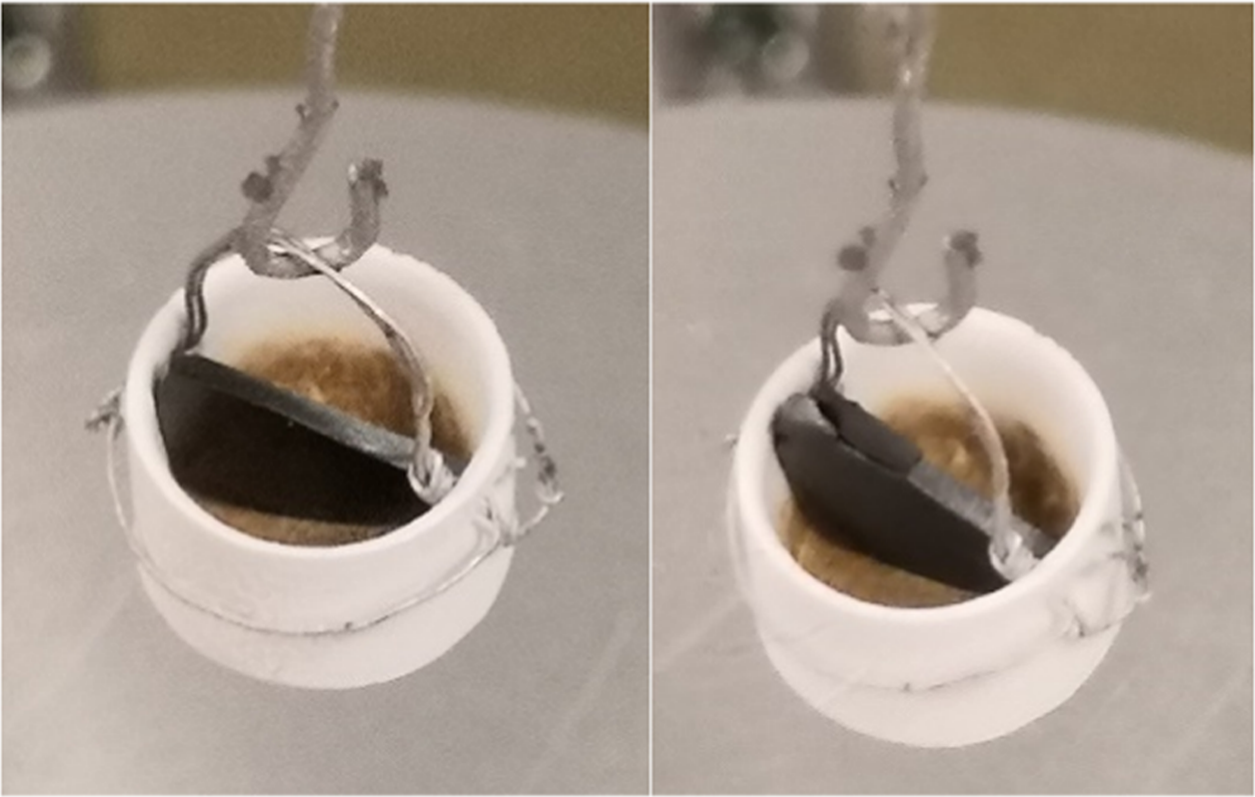
Dense tablet of CMTF8341 (left) prior to and (right) after 100 redox cycles at 1050 °C.

To study the effect of fundamental cation transport on the stability of perovskite OTMs, dense tablets of CMTFs (c.f. Figure 2) were first polished to have a flat surface prior to 100 redox cycles between 5% H_2_ ↔ 5% O_2_ at 1050 °C with 2 min for reducing and oxidizing and 2 min for purging in between. Note: such a high temperature would be extremely harsh for the materials investigated. After redox cycles, the microstructure and compositions of both the surface and cross-section of tablets were analyzed by SEM, optical microscopy, and EDS, as described in more detail in Section 2.6.

### 2.6 Phase and Microstructure Characterization

Pre- and postcharacterization was performed on the samples prior to and after redox cycling. The phase purity was investigated by X-ray diffraction using a PANalytical Empyrean diffractometer with Cu Kα radiation and a PIXcel^3D^ detector. With regard to the microstructure, the sieved materials were embedded in epoxy resin, and materialographic cross-sections were made using diamond-based grinding and polishing products. Field emission gun scanning electron microscopy (FEG-SEM) characterization was performed on a Nova NanoSEM650 (FEI Corp.). The analyses were performed in low vacuum mode (50–60 Pa H_2_O) in order to avoid electric charging of the surface without the necessity to coat the samples. Backscattered electron (BSE) images were taken that reflect the local density of the samples (high density induces high brightness). Elemental analysis and mapping were performed by using an X-Max50 (Oxford instruments) energy-dispersive spectrometer (EDS) attached to the FEG-SEM instrument. The surface microstructures of materials were also checked.

## 3 Results

### 3.1 Oxygen Loss as a Function of Oxygen Partial Pressure

**Figure 3:**
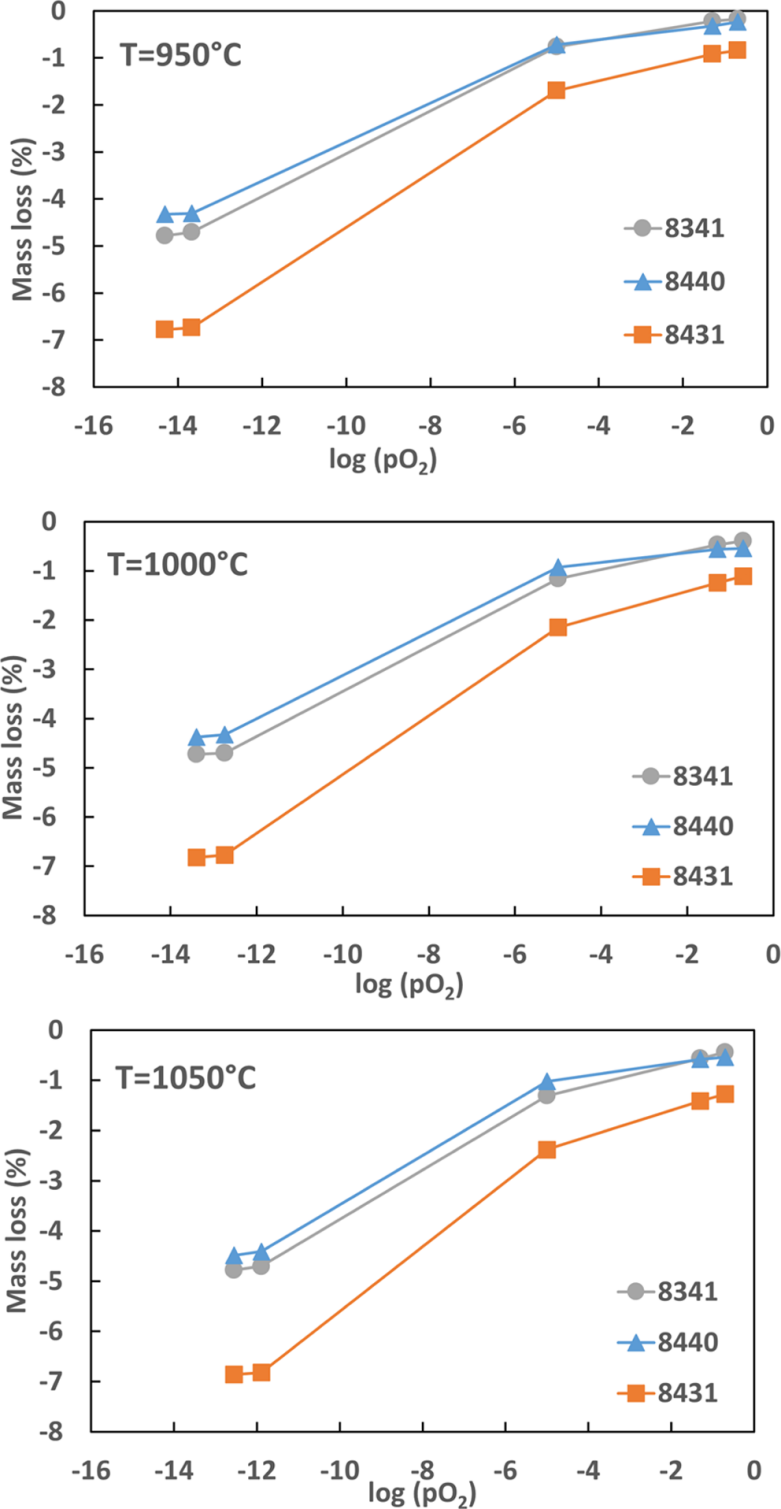
Mass loss of CMTF8341, CMTF8431, and CMTF8440 vs *p*O_2_ stepping at 950, 1000, and 1050 °C.

A series of oxygen mass loss tests have been performed for CMTF8341, CMTF8431, and CMTF8440 under equilibrium conditions, where the exact oxygen transfer (OTC) capacity was determined under certain *p*O_2_, as shown in Figure 3. The mass loss as a function of *p*O_2_ shows that CMTF8341 and CMTF8440 have comparable OTCs in the whole range of *p*O_2_, with values of 4.6 and 4.1 wt % at 950 °C, respectively. CMTF8431 has a slightly higher oxygen loss from oxidizing to inert conditions as compared to the other two. The difference is becoming more significant under reducing conditions such as 5 and 10% H_2_, with an OTC at 950 °C of ∼6 wt % for CMTF8431. All of these three perovskite systems also clearly show a chemical looping uncoupling (CLOU) effect, although the CLOU capacity (less than 1%) is much smaller as compared to their CLC capacity. The expected total OTCs for CMTF8314, CMTF8840, and CMTF8431 are 5.0, 5.7, and 6.4 wt %, respectively, when assuming the valence change of Mn from 4^+^ to 2^+^ and Fe from 3^+^ to 2^+^ (c.f. Table 1). The measured OTC indicates that CMTF8341 and CMTF8431 are close to being fully oxidized/reduced and reach more than 90% of its expected OTC, while CMTF8440 can only reach 72% of the expected capacity based on given assumptions. The mass loss as a function of temperature in air also shows that CMTF8431 with less Ti gives a higher OTC (ca. 1 wt %) as compared with the other two, indicating a higher CLOU effect given by the less stable structure toward reduction.

**Table 1:** Calculated OTC wt % with the Varying Reduction Valence of Mn from 4^+^ to 3^+^ for CLOU and Both of Mn and Fe to 2^+^ for CLC^a^

			exp CLOU			exp CLC		
	Mn^4+^ to Mn^2+^, Fe^3+^ to Fe^2+^ (wt %)	Mn^4+^ to Mn^3+^ (wt %)	950 °C	1000 °C	1050 °C	950 °C	1000 °C	1050 °C
CMTF8341	5.0	2.2	0.59	0.76	0.82	4.61	4.34	4.33
CMTF8431	6.4	2.9	0.86	1.04	1.11	5.94	5.71	5.59
CMTF8440	5.7	2.9	0.49	0.39	0.48	4.09	3.84	3.95

a Experimental CLOU is based on the mass loss between air and argon with CLC between air and 10% humidified H_2_.

### 3.2 Performance of Redox Cycles

**Figure 4:**
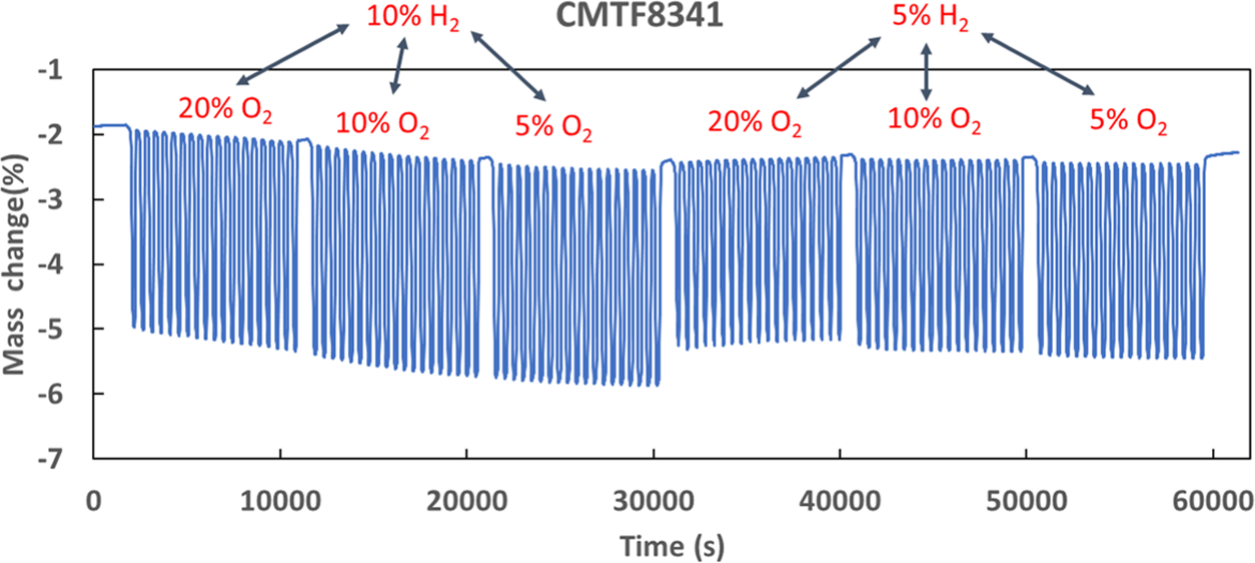
Typical TGA curve shows redox cycles of CMTF8341 under various oxidizing and reducing conditions at 950 °C.

A typical TGA curve for redox cycles has been plotted and is shown in Figure 4 using CMTF8341 as an example for showing test conditions of various cycles. The mass change corresponding to the change in the OTC was recorded as a function of time and redox cycles. Under the same reducing condition, varying the oxygen content did not influence the apparent OTC, while a less reducing atmosphere (5% H_2_) led to a slightly lower apparent OTC under the same oxidizing condition. This might be related to both the reduced driving force giving reduced kinetics for reduction and a lower OTC under given *p*O_2_.

**Figure 5:**
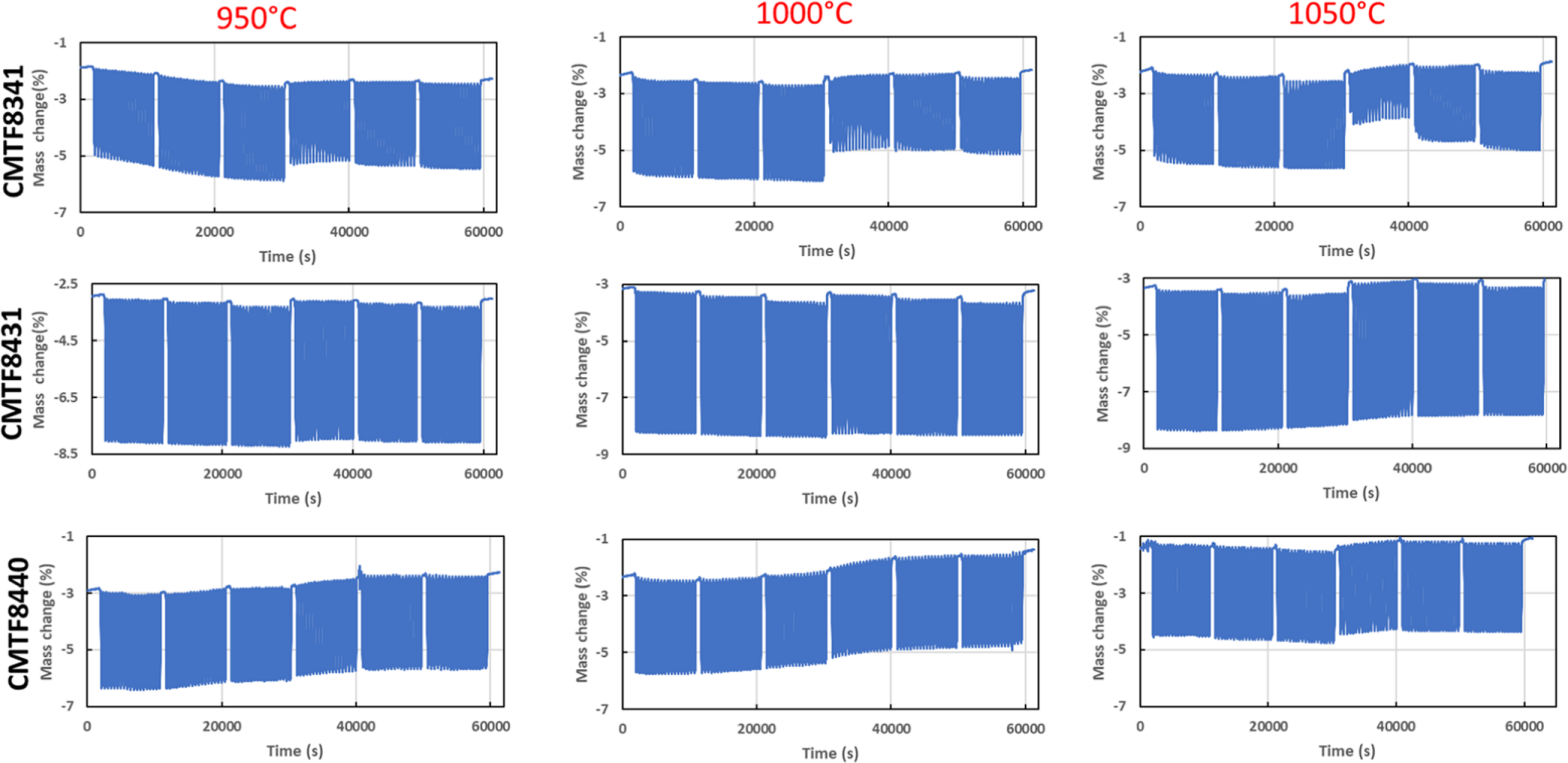
Comparison of redox cycles of CMTF series at 950, 1000, and 1050 °C.

Figure 5 shows the plotted TGA curves of redox cycles of three CMTF compositions, showing a good and stable performance according to OTC > 3 wt % at all temperatures. Among these three compositions, CMTF8341 has a relatively lower OTC, especially when a less reducing agent is used, while CMTF8431 has a much higher apparent OTC. These two compositions were further tested with different fuels, including CO and CH_4_. It is expected that CMTF8341 should be more stable, while CMTF8431 should be more reactive according to the concentration of Mn and Ti on the B-site.

**Figure 6:**
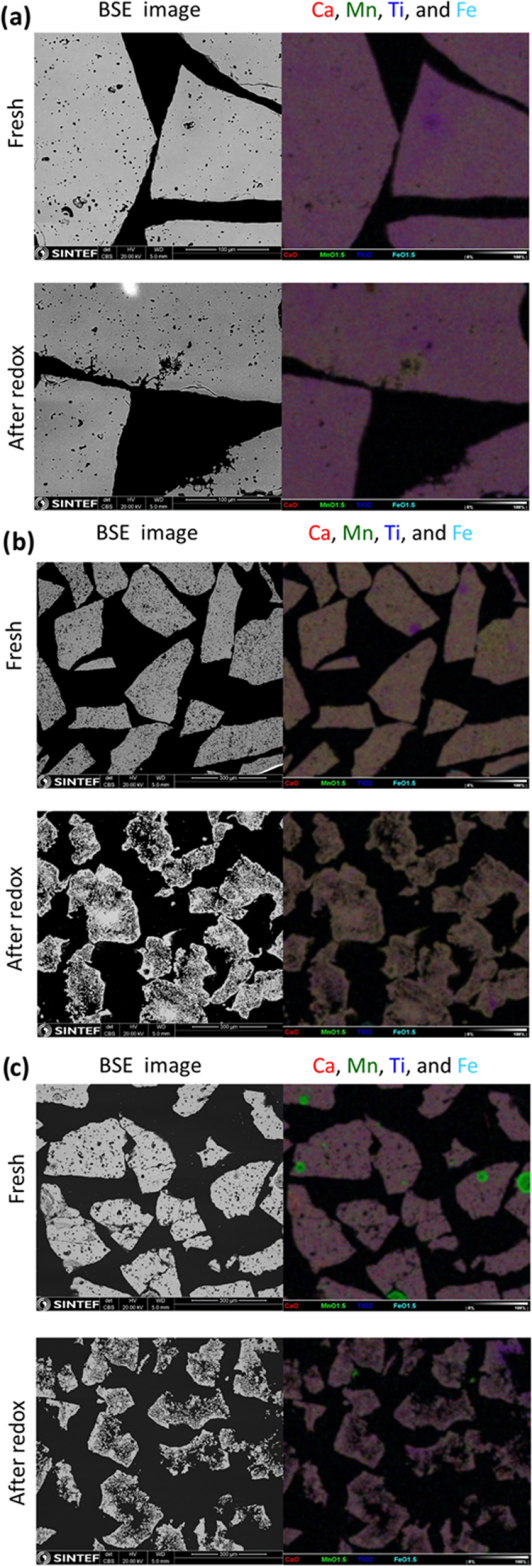
Comparison of the microstructure change prior to and after redox cycles for (a) CMTF8341, (b) CMTF8431, and (c) CMTF8440.

**Figure 7:**
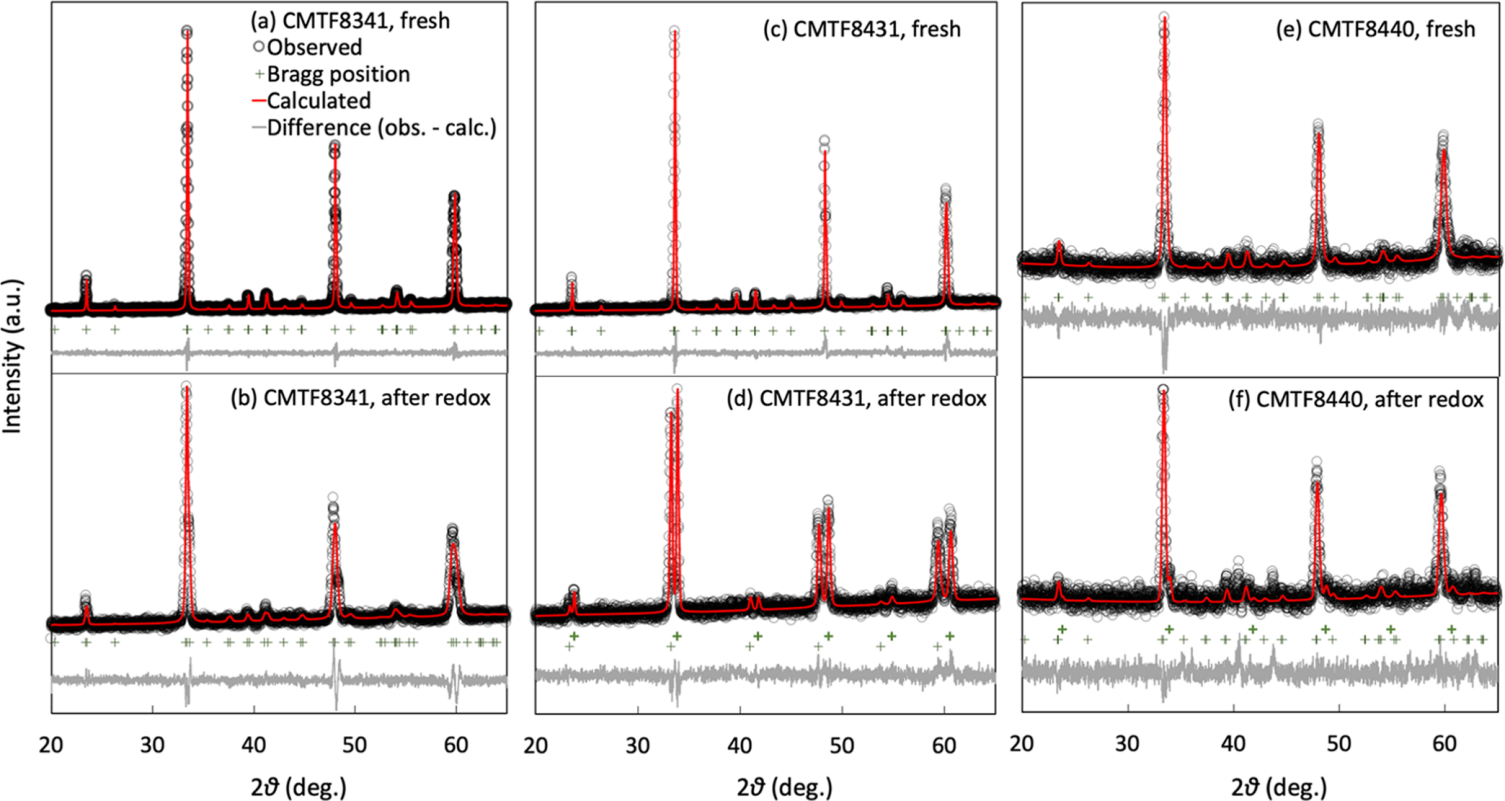
Comparison of the XRD pattern change prior to and after redox cycles for (a, b) CMTF8341, (c, d) CMTF8431, and (e, f)CMTF8440.

It is clear from the SEM images shown in Figure 6 that CMTF8341 has the most stable composition: no pores formed after deep redox cycles at high temperatures and also with a homogeneous composition. For CMTF8431 and CMTF8440, we can see that the materials become more porous especially at the center of the particles, but the compositions of the materials are mostly homogeneous. The highest apparent OTC during redox cycles is as expected for CMTF8431 with a lower Ti content than the other compositions. The XRD patterns shown in Figure 7 indicate that no new clear extra phases have been detected for CMTF8341 and CMTF8440 after deep redox cycling, while a phase separation into CaTiO_3_- and CaMnO_3_-rich phases can be noticed for CMTF8431. One should be aware that the redox conditions of the OCM particles in TG are much harsher than those in conventional reactor testing.

### 3.3 Fuel Type on the Redox Performance

**Figure 8:**
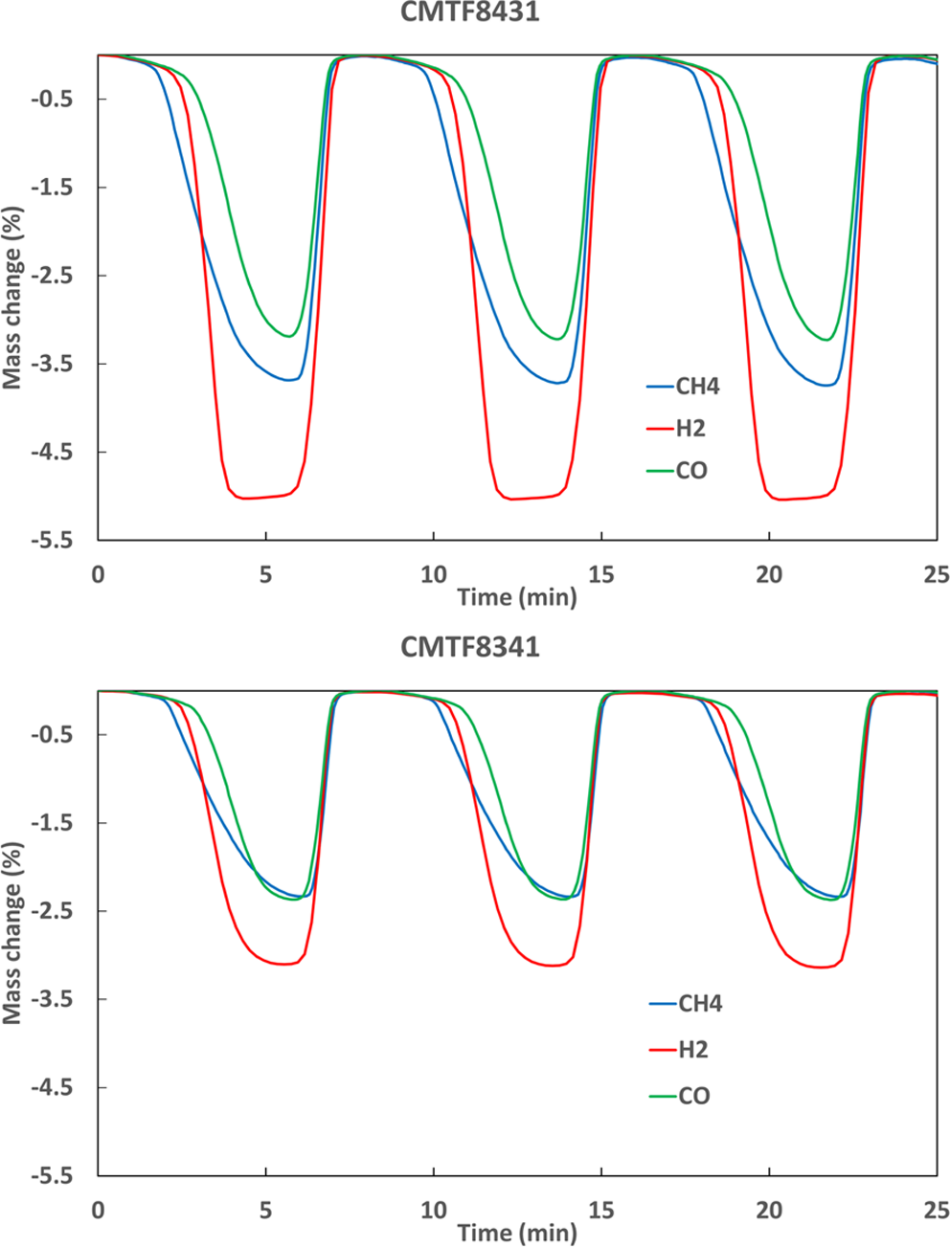
Comparison of redox cycles between different fuels (10% H_2_, 5% CO, and 10% CH_4_) vs 20% O_2_ at 950 °C.

**Figure 9:**
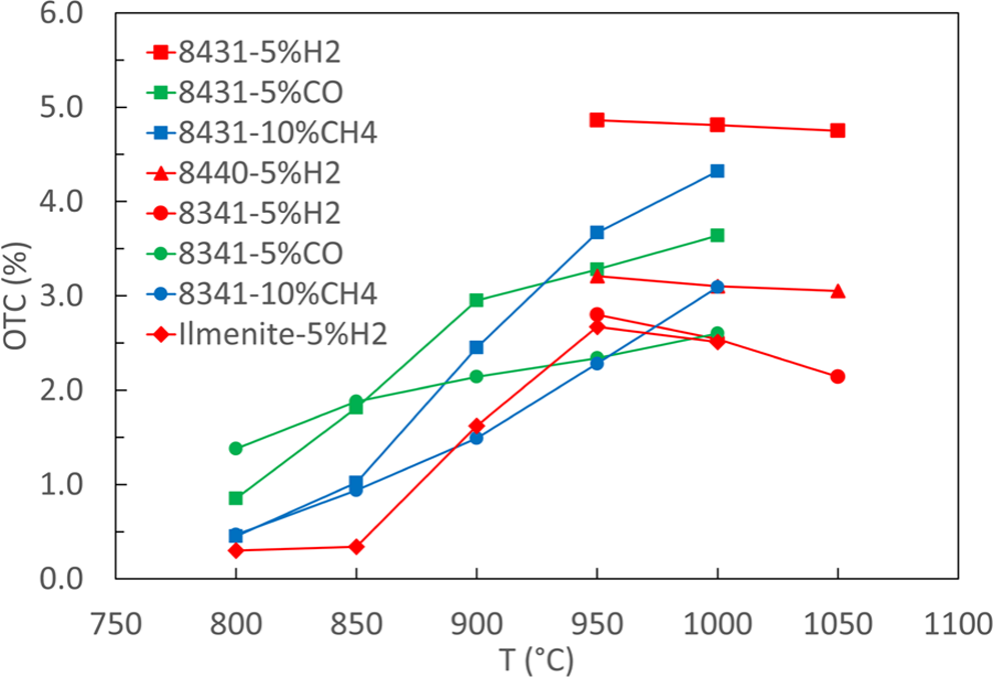
Oxygen transfer capacity with different fuels; the oxidizing condition is 20% O_2_. Ilmenite data from ref (6).

**Figure 10:**
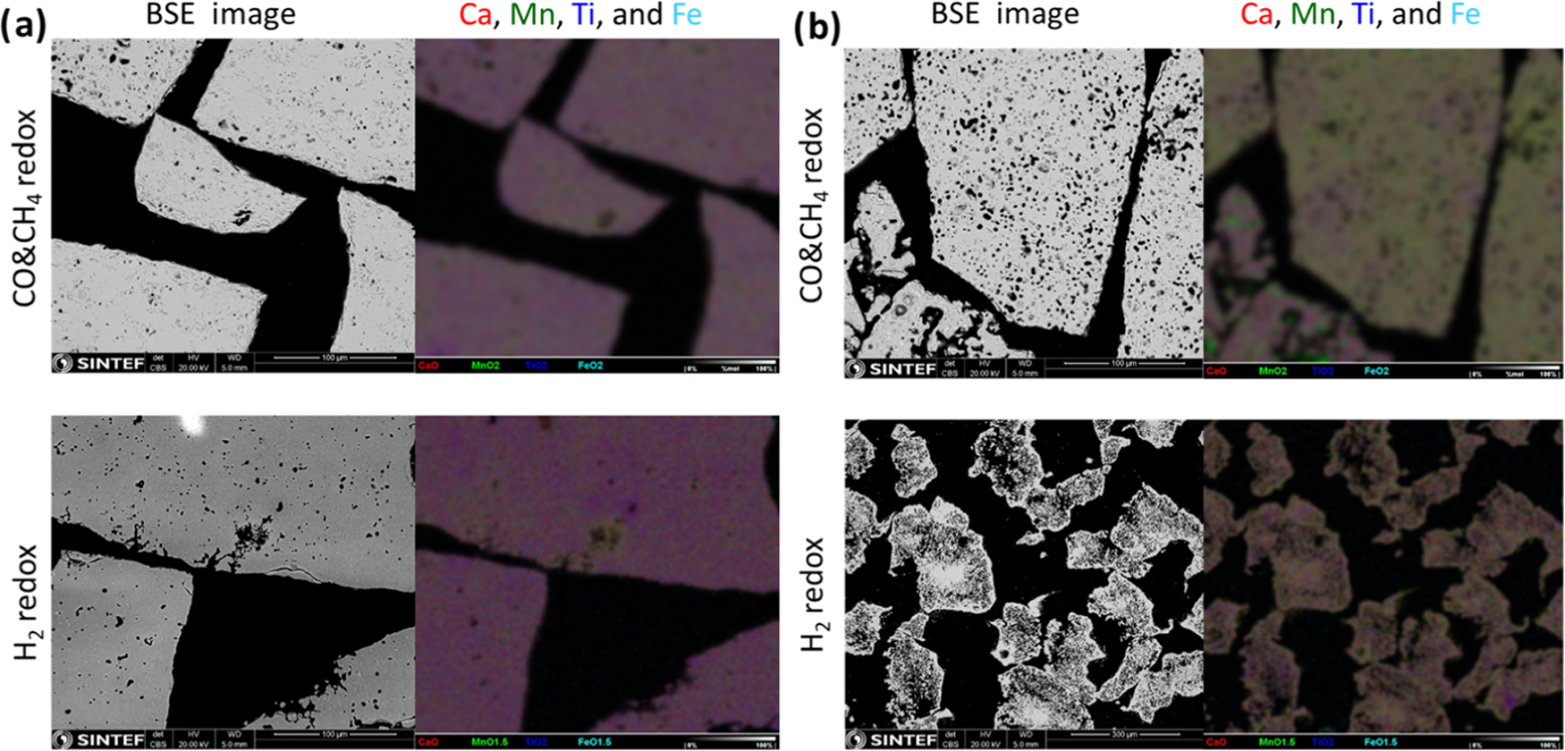
Microstructure change after redox cycling with different fuels for (a) CMTF8341 and (b) CMTF8431.

Humidified hydrogen has a fast reaction kinetics during redox cycling since it presents a more reducing condition than CO/CO_2_ mixtures and does not have the inert behavior as CH_4_ that often needs reforming to get active. Figure 8 clearly demonstrates that redox using H_2_ fuel gives a higher OTC than that with CO and CH_4_ for CMTF8431, while the difference is becoming smaller for CMTF8341. It is also interesting to see from the figure that oxidation curves are the same after reduction with different fuels, showing fast oxidation kinetics. Figure 9 compares the OTCs under various redox cycles, showing that CMTF-based perovskites have higher values especially at high temperatures as compared to the reference material ilmenite. Microsctructure characterizations by SEM, as shown in Figure 10, have shown that CMTF8341 does not develop either porosity or inhomogeneous distribution of compositions, no matter which fuel is used, while redox cycles of CMTF8431 using CO and CH_4_ as fuels show less degradation as compared to those under H_2_. As displayed in Figure 9, 2 min reduction in 5% humidified hydrogen gives an OTC of ca. 2.7 and 3 wt % for CMTF8341 and CMTF8440, respectively, indicating that Mn is at least reduced to the oxidation state 3^+^, corresponding to the calculated OTC of 2.2 and 2.9 wt % for CMTF8341 and CMTF8440, respectively, for such cases (c.f. Table 1). The OTC of 5 wt % for CMTF8431 for 2 min reduction in 5% humidified H_2_ is higher than the calculated 2.9 wt %, indicating a deeper reduction than the oxidation state of 3^+^, which is also in line with the lesser stability given by the lower Ti content in the structure. But the reduction of CMTF8314 (2.7 wt %), CMTF8440 (3 wt %), and CMTF834 (5 wt %) is still below the max reduction of Mn^4+^ to Mn^2+^ and Fe^3+^ to Fe^2+^ (Table 1), giving an expected total mass reduction of 5.0, 5.7, and 6.4 wt %.

**Figure 11:**
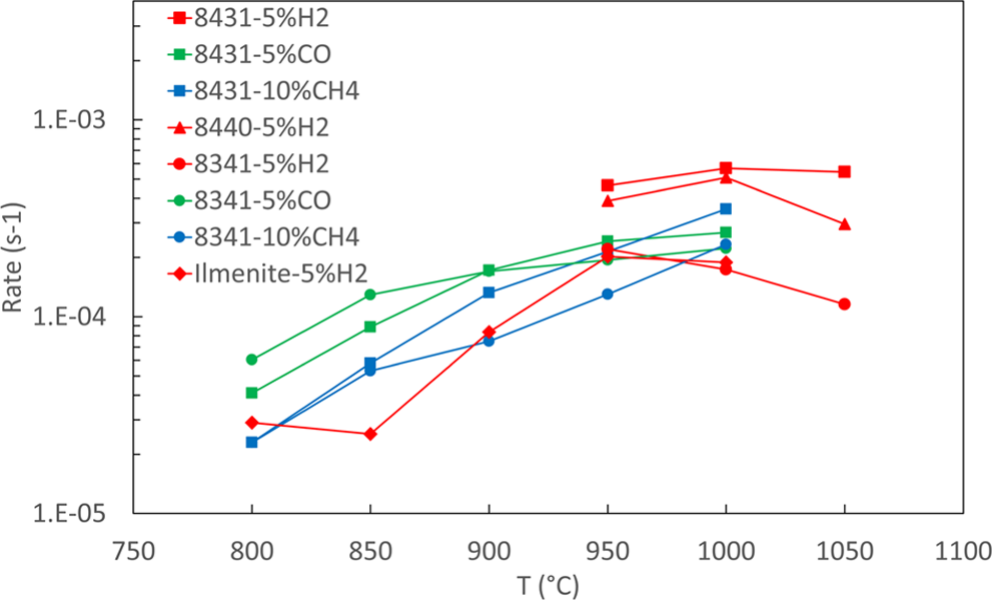
Oxygen reduction rate of CMTFs by using different fuels. The reference material is ilmenite.^6^

From the measured TGA curve, the oxygen release rate (or reduction rate) can be derived through
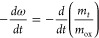
1where *m*_ox_ is the mass of the oxygen carrier at the fully oxidized state, and *m*_*t*_ is the measured mass change. By plotting the mass change vs time, the slope (i.e., oxygen release rate s^–1^) can be extracted. For the sake of comparison, we chose redox cycles between 5% H_2_ ↔ 20% O_2_, 5% CO ↔ 20% O_2_, and 10% CH_4_ ↔ 20% O_2_. Figure 11 shows that CMTFs have higher reduction kinetics as compared to conventional minerals such as ilmenite. Among CMTFs, the Mn-rich one (CMTF8431) has the highest reaction rate, while the Ti-rich one (CMTF8341) has the lowest reaction rate when using H_2_ and CH_4_ as the reducing agents. It seems that the reduction rate levels out for CMTF8431 and decreases as the temperature increases.

### 3.4 Stability due to Cation Diffusion

The stability of the perovskite structure (ABO_3_) is often related to the cation diffusion in the crystal. Under redox cycling, oxygen diffuses inward and outward, resulting in opposite diffusion of the cation according to the Gibbs–Duhem rule *n*_A_*d*μ_A_ + *n*_B_*d*μ_B_ + *n*_O_*d*μ_O_ = 0, where *n* is the concentration, and μ is the chemical potential. If the diffusivity of cations is too fast, the material may demix or even decompose to a certain extent.^15,16^

**Figure 12:**
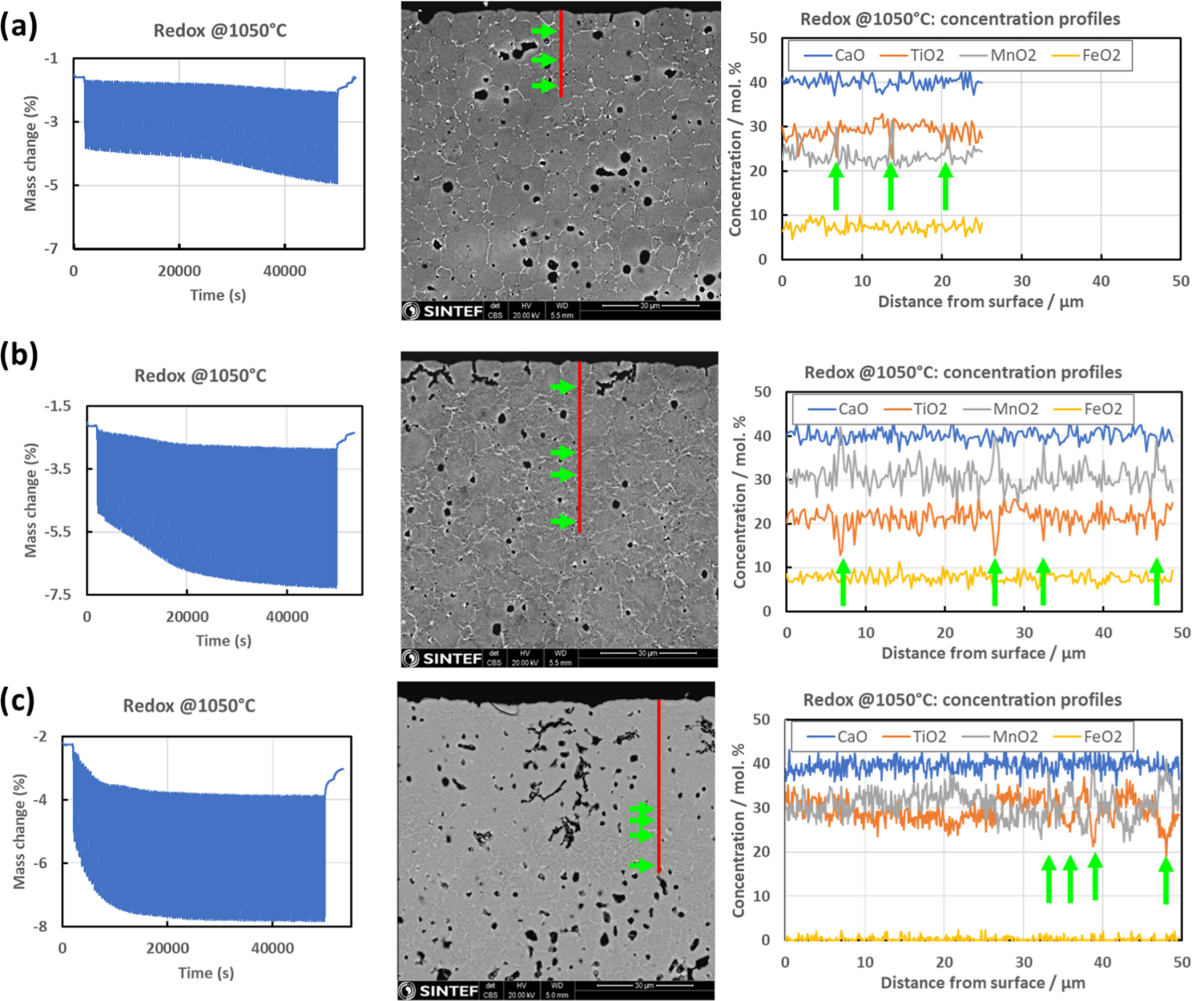
From left to right: TGA curve for 100 redox cycles at 1050 °C, SEM images of the cross-section of the oxygen carriers after redox, and depth profiling of cation compositions after 100 redox cycles (the red line on the SEM image is the direction of EDS line scanning): (a) CMTF8341, (b) CMTF8431, and (c) CMTF8440.

Figure 12 shows the redox cycle performance of the dense tablet OCMs at 1050 °C. From the TGA curves (left side of the figure), it is quite evident that the level of the OTC increases as the cycling goes on and reaches approximately 3 wt % for CMTF8341, 4.5 wt % for CMTF8431, and 4 wt % for CMTF8440, which are slightly lower than the values obtained for the powder samples, as shown in Figure 9. Considering the dense tablet samples used for such redox cycling testing, the investigated CMTF-based perovskite oxygen carriers must possess fast oxygen transport in the bulk and fast surface exchange at the surface. From the SEM post-characterizations of the cross-sections after redox cycling experiments, only small pores have formed under such harsh redox cycling conditions. Moreover, EDS line scanning shows a homogeneous distribution of elements along the depth from the surface to the bulk for all of the three investigated compositions. Cation demixing, cation segregation, or material decomposition has not been observed for the investigated materials, showing a super stable structure. One point that should be mentioned here is that only Mn enrichment/Ti depletion along grain boundaries has been observed, as marked by green arrows.

**Figure 13:**
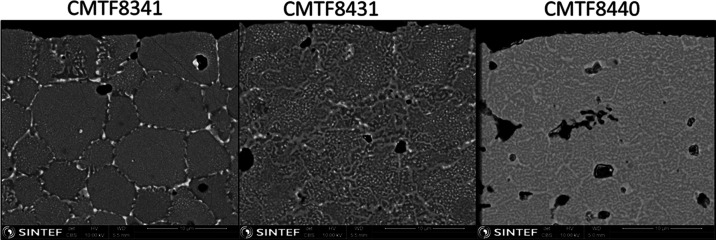
Comparison of the surface microstructure of CMTF8341, CMTF8431, and CMTF8440 changing after redox cycling at 1050 °C.

Figure 13 shows the surface microstructure change of these three compositions upon redox at 1050 °C, which is the highest measured temperature. It is quite clear from the SEM images that more pores are formed along the grain boundaries, leading to weakening of the material strength.

## 4 Discussion

Reduction of CMTF by removing oxygen will often lead to an expansion by compensating bigger reduced B-site cations (eq 2 using the Kröger–Vink notation). Keeping the material inside the Goldsmith factor^17^ of the perovskite space under both oxidizing and reducing conditions will be a good starting point to avoid detrimental phase changes during redox cycles. The oxygen mobility in polycrystalline materials is also expected to be higher in structures with a high symmetry, but it is also influenced by preferences for defect formation given by the elemental composition.^18,19^ The calculations depicted in Table 2 show that under reducing conditions, CMTF8431 has the lowest Goldsmith factor, indicating that the structural stability is lower than the Ti-rich compounds. The obvious way should then be to precipitate the biggest cation, which in this case is Fe^2+^. On the contrary, enrichment of Mn–O along grain boundaries has been observed (c.f. Figure 12), which could have been formed according to eq 3, showing alternative reduction of the perovskite by oxygen removal compensated by production of metal vacancies and forming a secondary phase.

**Table 2:** Calculated Goldsmith Factors for the Different Perovskites Using Shannon’s Ionic Radius for the Different Elements^a^

	Ti^4+^Mn^4+^Fe^3+^	Ti^4+^Mn^3+^Fe^3+^	Ti^4+^Mn^2+^Fe^2+^
CMTF8341	0.9779	0.9687	0.9445
CMTF8431	0.9826	0.9703	0.9408
CMTF8440	0.9847	0.9724	0.9509

a The perovskite space is often given to be between 1.05 and 0.85 but is preferable between 1 and 0.95 for cubic phases.


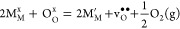
2where M_M_^x^ is the B-site, M_M_^′^ is a M^3+^ cation on the B-site, O_O_^x^ is the oxygen site, and v_O_^••^ is an oxygen vacancy.
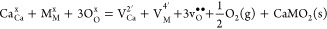
3where Ca_Ca_^x^ is the A-site, V_Ca_^2′^ and V_M_^4′^ are formed vacancies on A- and B-sites, respectively, and the other terms have their usual meanings.

The general trends for three CMTF compositions are the composition with a low amount of Ti, i.e., CMTF8431, has the highest redox performance, e.g., a high oxygen transfer capacity and a fast reduction rate, but the stability is lower under harsh redox cycles, while there is not so much difference for the other two compositions, i.e., a reasonably good performance but a higher stability. The lower Ti content in this case also gave a lower structural stability, as shown by the porous particles and phase separation formed after long-term cycle testing, as shown by SEM and XRD measurements. A higher CLOU capacity was also found on the CMTF8431 composition (c.f. Table 1). Increasing the Ti content gives a lower OTC but induces less physical stress during redox cycles and consequently a higher stability. CMTF8341 seems to be much more stable than CMTF8440, indicating that the cation diffusion is more restricted having Fe in this perovskite, even though the highest temperature also gave some Mn precipitation into the grain boundaries.

When looking back at the reduction rates for 5% H_2_ for these three compositions, as shown in Figure 11, CMTF8431 is still the fastest followed by CMTF8440, while CMTF8341 is the slowest. The fast reduction rates in CMTF8431 and CMTF8440 are most probably due to the high Mn content with low redox enthalpies indicating low binding energies to oxygen.^20^ However, substitution of Fe in CMTF8341 apparently did not achieve the same catalytic effects as those in Mn, probably due to the formation of Fe^4+^. Considering both the fast reduction rate and the high oxygen loss, it is no surprise that CMTF8431 has the highest measured OTC among these three under similar redox cycling.

When it comes to the stability under redox cycles, it is clearly shown that CMTF8431 particles form more pores than the other two compositions (c.f. Figure 6 and Figure 10), even the phase separation from XRD analyses (c.f. Figure 7), while the other two are very stable under such conditions, especially for CMTF8341. For a dense ceramic tablet under redox cycles, phase separation is only observed on the surface at a high temperature of 1050 °C. When checking the detailed composition depth profiles in Section 3.4, it is clearly shown that most of the elements are uniformly distributed from the surface except segregation of Mn along grain boundaries. This is probably due to the fast cation diffusivity of Mn, observed among a series of B-site cations in perovskites.^21^ It can easily lead to the formation of the reduced CaO–MnO phase on the grain boundaries following eq 3, which can get reoxidized and form the perovskite CaMnO_3_. Cation diffusion is a thermal-activated process with much higher activation energies than anion diffusion such as oxygen. The CMTF perovskite is a very promising system as the OCM based on abundant materials with a low cost. The CMTF composition range also gives the possibility to tailor for different windows of operation. It is also promising, given its high activity without the use of less environmentally friendly cobalt, as used in well-known BSCF and LSCF.

## 5 Conclusions

In the present work, three compositions of Ca(Mn–Ti–Fe)O_3_-based perovskite oxygen carriers have been developed for working up to 1050 °C in the CLC mode. The less stabilized sample with a low Ti content, CaMn_0.5_Ti_0.375_Fe_0.125_O_3−δ_ (CMTF8431), shows the best performance of redox cycling from the TGA measurements with an OTC up to 5.6 wt % at 1050 °C. On the other hand, Ti-rich CaMn_0.375_Ti_0.5_Fe_0.125_O_3−δ_ (CMTF8341) does not show any critical degradation of the microstructure under redox cycling at 1050 °C; it only shows some degree of segregation of Mn to the grain boundaries. For the CLC purpose, the rate of oxygen release during the residence time in the fuel reactor is of importance since it affects the combustion efficiency and reduces the need for oxygen polishing. In the countercurrent flow CLC design, a higher degree of the OTC of CMTF8431 can be utilized for combustion up to 5.6 wt % O_2_ as compared to the cocurrent flow where the OTC utilization needs to be limited to the CLOU effect ca. 1 wt % for achieving full combustion. The perovskite’s flexible composition gives the possibility to produce long-term stable oxygen carriers with very long lifetimes compared to redox systems investigated so far and adjust them to the different process conditions in different types of chemical looping technologies.
